# The moral responsibility of governments and individuals in the context of the coronavirus pandemic

**DOI:** 10.1177/1403494821990250

**Published:** 2021-02-06

**Authors:** Jessica Nihlén Fahlquist

**Affiliations:** Centre for Research Ethics and Bioethics, Uppsala University, Sweden

**Keywords:** Coronavirus, pandemic, moral responsibility, ethics

## Abstract

**Aim:**

Not only is the coronavirus pandemic about science and facts, it also raises a number of ethical questions. Some of the most important questions in this context are related to responsibility. First, what is a government’s primary responsibility? Second, how should both the government and individuals consider personal moral responsibility in this context?

**Method:**

This paper uses conceptual and normative analysis to address responsibility in the context of the pandemic. The paper also refers to reports published by the German Ethics Council, the Malaysian Bioethics Community and the Swedish National Council on Medical Ethics.

**Results:**

The primary responsibility of governments is to create a balance between individual values and rights, one hand, and the health of the population, on the other. There are good reasons to conceive of individual responsibility as a virtue, having to do with the development of crucial character traits and habits. The responsibility of governments is connected to individual responsibility through the values of trust and solidarity.

**Conclusions:**

Governments need to communicate clearly (a) how they balance conflicts between collective health and individual rights and values and (b) what the chosen strategy entails in terms of collective and individual responsibility. Success requires attention to ethical values from all involved. Individuals will need to develop new character traits to help manage this pandemic and to prevent new ones. Governments must facilitate the development of such character traits by building trust and solidarity with and among citizens.

## Introduction

The coronavirus pandemic has caused many fatalities and hospitalisations, and we do not know how the crisis will develop as of yet (January 2021). Organisations such as the German Ethics Council (GEC) [1], the Malaysian Bioethics Community (MBC) [2] and the Swedish National Council on Medical Ethics (SMER) [3] have acknowledged that the pandemic raises questions not only about the efficacy of various strategies but also difficult ethical questions.

Some of the most important ethical questions that have arisen in the context of the coronavirus pandemic concern moral responsibility. This paper focuses on three such questions. First, what is a government’s primary responsibility? Second, how should both the government and individuals consider personal moral responsibility in the context of the pandemic? Third, what is the connection between the government’s responsibility and the responsibility of individuals?

The paper is structured as follows. I begin with a discussion of risk-management strategies and the role of ethical values in risk management. Then, I explain why the primary responsibility of governments is to make public-health decisions that create a balance between individual rights, on the one hand, and the health of the population, on the other. Next, I address why conceiving of and promoting individual responsibility as a virtue provides another potential approach to combatting the pandemic. Finally, I explain why the values of trust and solidarity connect the responsibility of governments to individual responsibility.

## Risk management and ethical values

Governments in different countries, and sometimes within countries, have chosen different strategies. Desvars-Larrive et al. [4] classify countries’ responses to the pandemic into one of three categories: (a) laissez-faire, (b) herd immunity or (c) aggressive. The laissez-faire strategy involves few measures, if any. The herd immunity strategy consists of relying on voluntary measures. Finally, aggressive strategies are based on the implementation of a wide range of stringent interventions, some of which entail a limitation of civil rights [4,5]. To give just a few examples, the governments of China, Hong Kong and Taiwan quickly introduced strict rules regulating quarantines and lockdown, which illustrates the more aggressive strategy. In Chile and Argentina, policy and army forces enforced lockdowns [6]. Many African and European states initially took a more aggressive approach to the pandemic, including measures such as strict lockdown, curfews and the shutting down of cultural and economic activities. Some countries relaxed the rules during the summer but went back to a more aggressive approach in October, as it became clear that there was increased spread of the infection again [7,8].

Other countries, including Brazil and the USA, have taken an approach that privileges individual liberty over public health. Both Prime Minister Bolsonaro and President Trump have been heavily criticised for being too laissez-faire, for reacting too late and for endorsing questionable treatments to combat the disease [9].

Sweden and Norway issued voluntary but strong recommendations, and it has been argued that the former adopted a herd immunity approach [4], although this has been denied by the Swedish state epidemiologist Anders Tegnell. He claims that the herd immunity approach is questionable and that the goal has been to slow down the spread of contagion and to facilitate management of the health-care system [10]. Nevertheless, the Swedish and Norwegian approaches were not as strict as most other countries. The initial response by the UK government was similar to the Swedish strategy, introducing stricter regulations after some time had passed, for which it was criticised [11].

## Responsibility of governments: balancing individual rights and the collective good

Experts have primarily communicated about the coronavirus pandemic in numerical terms, for example describing the importance of ‘flattening the curve’. This is consistent with research showing that experts consider risk to be acceptable if the benefits outweigh the harms, and risk is calculated at the population level. In contrast, laypeople tend to take other values into consideration, such as whether a risk was voluntary and whether risks and benefits are distributed fairly. Against this background, it is important that communicators take these ethical values into account [12]. Furthermore, the balancing of those values should be made explicit. There are many examples where experts and leaders state that the reason for, for example, not prohibiting all cultural events is that there is not enough evidence that this is effective [13]. This could instead be described as partly a matter of what appears to be effective and partly about the value of quality of life and the right to maintain a certain measure of autonomy.

Experts can make risk communication more effective as well as ethically legitimate by taking the layperson’s approach into account [14,15]. As mentioned in a report by the GEC, democratic legitimacy requires that public-health policy should not be delegated exclusively to scientists, but rather should take values into account. Given the complexity of making public policy decisions concerning the pandemic, ‘a showdown between public health imperatives and civil liberties appears inevitable’ [1].

Governments therefore have to walk a fine line between protecting the collective good and upholding ethical values. This balance is crucial due to the power imbalance between authorities and laypeople [16]. As the GEC states, governments should make sure that the fundamental rights of individuals are upheld, even in cases where a utilitarian approach to ‘save as many lives as possible’ is also a duty. It is the ‘democratic responsibility’ of society as a whole to decide how to respond to scientific findings, instead of delegating policy decisions to scientists. The core ethical issue requires taking the right measures to ‘sustainably safeguard a high-quality and effective healthcare system whilst, at the same time, averting or mitigating the serious adverse consequences of these measures for people and society’ [1]. The MBC also identifies ‘the tensions between public health interests and personal rights and freedom’ as a key ethical issue [2].

The main values at stake can be evaluated using the four principles of medical ethics: autonomy, non-maleficence, beneficence and justice [17]. Although they are neither all-encompassing nor above criticism, these principles are useful for this analysis. For example, these principles help us see that the collective good relates more to beneficence and justice. The values often espoused by laypeople involve questions relating to autonomy and non-maleficence.

### Beneficence and justice

The principle of beneficence can be described as ‘a statement of moral obligation to act for the benefit of others’ [17]. As public health focuses on the good of the population, this principle could be seen as requiring the government to act for the benefit of the population instead of focusing on benefit to a specific individual. Protecting and promoting the health of the population is the rationale of public health, which means that the principle of beneficence is a dominant principle. Managing the coronavirus pandemic requires the protection of as many people as possible from infection, which has the added benefit of easing pressure on health-care systems. The principle of beneficence applies in the context of the coronavirus to the prevention of harm. Simultaneously preventing harm to vulnerable populations, the population at large and individuals is a challenging task.

The principle of justice involves ‘fair, equitable, and appropriate treatment in light of what is due or owed to persons’ [17]. This encompasses the notions that (a) everyone should be treated equally and that (b) resources should be distributed fairly. This means that everyone should have an equal chance at good health and a long life. This relates to prevention of harm because a core problem of justice involves how to make decisions balancing the needs of patients in urgent need of health care and people who suffer from, for example, postponed treatments.

Some people will be harmed as a consequence of protecting others. For example, the resources needed to address the pandemic led to medical procedures being cancelled or delayed. In Sweden, one of the major hospitals cancelled all non-urgent surgery due to a coronavirus outbreak [18]. Additional unintended side effects are the increased risk of partner abuse and mental-health issues [19].

### Autonomy and non-maleficence

Individual rights entail questions relating to the values of autonomy and non-maleficence. Autonomy is considered closely related to freedom of choice. Beauchamp and Childress state that respect for autonomy means acknowledging the rights of individuals to make choices and to ‘take actions based on their personal values and beliefs’ [17]. A more nuanced approach considers autonomy on a spectrum between ‘shallow autonomy’ and ‘deep autonomy’. For example, seat-belt requirements and stop signs would be considered infringements on autonomy if it is understood merely as the freedom to act (i.e. ‘shallow autonomy’). In contrast, the value of ‘deep autonomy’ could be conceptualised as the value of making one’s own important life choices, assessed over a longer period of time, involving ‘reflection on the values by which one’s life will be structured’ [20]. It entails respect for an individual’s choices, but also for their capacity for conscious reflection upon these values. From this perspective, seat-belt laws are not infringements of autonomy because individuals can choose not to abide by the law [20,21].

Individual autonomy has been challenged during the coronavirus crisis, as people have been prohibited or discouraged from going outside, visiting relatives and attending cultural events and social gatherings. Each government has had to choose between a laissez-faire or herd immunity strategy that maintains as much autonomy as possible and an aggressive strategy that prioritises the health of high-risk groups. Regardless of the specifics, every national strategy has had to take a position along this continuum.

The principle of non-maleficence entails an obligation to refrain from harming others [18]. The connection, and potential conflict, between autonomy and the right not to be harmed was described by Mill, who argued that we are allowed to do anything we want as long as it does not infringe on anyone else’s right not to be harmed [22]. Not following the rules during the coronavirus pandemic can clearly cause harm to others as a consequence.

Laypeople generally value voluntariness and autonomy in risk taking. However, they also value fairness in risk–benefit distributions [23]. In light of this, infringements of the right to autonomy may be considered acceptable if the underlying reason is to protect vulnerable people from being exposed to fatal risks. Against this background, the challenge for governments is to communicate the way in which autonomy and non-maleficence are being balanced against beneficence and justice.

## Individual responsibility

Decreasing the spread of the pandemic also requires that individuals take personal responsibility. As the GEC states, each individual has a responsibility to know that one’s own decisions necessarily have an impact on other people [1].

The pandemic resembles problems such as climate change and antibiotic resistance in that they are not likely to be solved or managed unless both states and individuals take actions. The distribution of responsibility between governments and individuals is one of the main ethical issues discussed in relation to climate change and antibiotic resistance. State action is crucial, but unless there are also behavioural changes among the individuals making up the population at large, long-term change is unlikely to occur. It is crucial for the current pandemic, but it will be even more important to prevent similar challenges in the future.

Regulations are important but inadequate. Studies show that rules are not enough to change people’s behaviour, but change of social norms, habits and so on is also necessary. For example, lifestyle changes that are needed to reduce obesity are unlikely unless sustainable developments of habits and norms are encouraged [24]. These changes have to be not merely initiated but also maintained. As described by Maio et al. [25], research distinguishes between downstream and upstream interventions. Downstream interventions include, for example, information campaigns. Upstream interventions focus on the environment and long-term change of social norms in order for desired habits to flourish, shaping conditions that ‘promote and sustain desired habits’ [25,26].

A useful way of conceiving this kind long-term change comes from virtue ethics. Whereas consequentialism and deontological ethics focus on actions and individual decisions, virtue ethics departs from the entirety and complexity of people’s lives. It emphasises the importance of human beings gradually evolving virtues, which could be facilitated by environment, social context and role models. Just like Jamieson argues in relation to environmental problems, there are contexts where even utilitarians should be concerned with virtues because that will be more effective [27].

Conceiving responsibility as a virtue implies requiring individuals to be capable of navigating the complexity of relevant values and principles in order to be able to ‘respond to a plurality of normative demands’ [28]. The pandemic increases the complexity of responding to normative demands, with individuals having to find new ways to organise work and parenting, and to care for the elderly without compromising their health. The longevity of the crisis calls for the cultivation of certain character traits and habits, such as an increased compassion for others.

However, the expectation that individuals will take responsibility for collective problems must be connected to individual contexts [15]. Having secure, well-paid employment with the opportunity to telework makes it easier to adopt new ways of living. However, insecure employment with required in-person attendance and daily use of public transport makes it more difficult. Normative demands vary widely from person to person, which influences the ability to develop personal responsibility as a virtue. Some individuals who would choose to be conscientious may simply be unable to follow the rules. That said, governments can encourage the development of responsibility as a virtue by building trust and solidarity.

## The connection between governmental and individual responsibility

Virtues can be influenced through governmental action. Two values are particularly important for facilitating a positive relationship between the government and individuals: trust and solidarity. As we have seen, solving societal problems requires both governmental and individual responsibility. Working together requires trust and solidarity between governments and individuals.

### Trust

For people to be willing to take responsibility to develop the habits necessary for managing a pandemic, they need to trust their government. Trust is relational and requires that the trusted party be worthy of trust. However, trust in public-health officials has decreased in the past few years. This has led to parents refusing to have their children vaccinated. There are many reasons why people mistrust vaccination, including concerns about safety and efficacy, religious beliefs and social norms. Authorities overstating the benefits or understating the risks of vaccination have jeopardised trust. For example, during the H1N1 mass vaccination campaign in Sweden, the government called the vaccine safe. However, the vaccine caused some teenagers to develop narcolepsy. Failure to communicate the risks of new vaccines, compared to old ones, can diminish trust between laypeople and experts [16].

Vaccination can be seen as taking responsibility for protecting others as well as oneself. Forcing people to protect others is unlikely to encourage virtuous behaviour. As Dawson et al. put it, ‘You can compel action, but not trust’. Trustworthiness in those given responsibility requires action, and if authorities are open and honest with their information, trust is more likely to be maintained [29]. The MBC states that trust is strengthened by transparency and inclusiveness [2].

### Solidarity

Interestingly, the GEC’s use of the concept of solidarity is very close to that of responsibility as a virtue. It states that ‘[s]olidarity means the willingness to take pro-social action on the basis of relevant common ground that demands something from the person who is prepared to show solidarity’. It is based on ‘human compassion’ and a ‘basic feeling of togetherness’, but must also be translated into actions [1].

SMER conceptualises responsibility as being a part of solidarity: ‘It is important to support those individuals who risk being hit particularly hard by infection or countermeasures, while also emphasising individual responsibility for the choices they make in their daily life’. Voluntary recommendations (instead of mandatory rules) can contribute to trust and ‘a sense of joint responsibility’ [3].

Dawson and Verweij distinguish between *rational* and *constitutive* solidarity. The former is based on the idea that a threat to one individual is also a threat to everyone, which entails a focus on collective societal preparation for, and prevention of, pandemics. Rational solidarity has provided the justification for many of the measures taken during the coronavirus crisis. Constitutive solidarity describes the voluntary action of people to help others, that is, taking responsibility. Citizens in countries encouraging social distancing without enforcing it could be seen as encouraging this kind of solidarity [30]. It is difficult to know exactly what effect policies such as police enforcement of social distancing have on people’s feelings of solidarity. If the crisis can be resolved soon, countries that focus on rational solidarity and promote a sense of obligation to others may find this moderate approach to be sufficient. However, the longer a crisis such as this one lasts, the greater the need for people to feel connected, which creates an even greater need for individuals to develop responsibility as a virtue.

## Conclusions

To overcome the pandemic, both governments and individuals need to take responsibility. Governments must communicate clearly (a) how they balance conflicts between collective health and individual rights and values and (b) what the chosen strategy entails in terms of collective and individual responsibility. These tasks require an open public discourse about the values involved. While experts can provide numbers and facts, individuals need to be involved in determining which decisions are made and how decisions are made. Success requires attention to ethical values from all involved. Individuals will need to develop new character traits to help manage this pandemic and to prevent new ones. Governments must facilitate the development of such character traits by building trust and solidarity with and among citizens.
